# Fine-Mapping and Phenotypic Analysis of the *Ity3 Salmonella* Susceptibility Locus Identify a Complex Genetic Structure

**DOI:** 10.1371/journal.pone.0088009

**Published:** 2014-02-04

**Authors:** Rabia T. Khan, Kyoko E. Yuki, Danielle Malo

**Affiliations:** 1 Department of Human Genetics, McGill University, Montreal, Québec, Canada; 2 Complex Traits Group, McGill University, Montreal, Québec, Canada; 3 Department of Medicine, McGill University, Montreal, Québec, Canada; Duke University Medical Center, United States of America

## Abstract

Experimental animal models of *Salmonella* infections have been widely used to identify genes important in the host immune response to infection. Using an F2 cross between the classical inbred strain C57BL/6J and the wild derived strain MOLF/Ei, we have previously identified *Ity3* (Immunity to Typhimurium locus 3) as a locus contributing to the early susceptibility of MOLF/Ei mice to infection with *Salmonella* Typhimurium. We have also established a congenic strain (B6.MOLF-*Ity/Ity3)* with the MOLF/Ei *Ity3* donor segment on a C57BL/6J background. The current study was designed to fine map and characterize functionally the *Ity3* locus. We generated 12 recombinant sub-congenic strains that were characterized for susceptibility to infection, bacterial load in target organs, cytokine profile and anti-microbial mechanisms. These analyses showed that the impact of the *Ity3* locus on survival and bacterial burden was stronger in male mice compared to female mice. Fine mapping of *Ity3* indicated that two subloci contribute collectively to the susceptibility of B6.MOLF-*Ity/Ity3* congenic mice to *Salmonella* infection. The *Ity3.1* sublocus controls NADPH oxidase activity and is characterized by decreased ROS production, reduced inflammatory cytokine response and increased bacterial burden, thereby supporting a role for *Ncf2* (neutrophil cytosolic factor 2 a subunit of NADPH oxidase) as the gene underlying this sublocus. The *Ity3.2* sub-locus is characterized by a hyperresponsive inflammatory cytokine phenotype after exposure to *Salmonella*. Overall, this research provides support to the combined action of hormonal influences and complex genetic factors within the *Ity3* locus in the innate immune response to *Salmonella* infection in wild-derived MOLF/Ei mice.

## Introduction

Despite reduction of morbidity as a result of the development of antibiotics and vaccines, infectious diseases are still among the leading causes of morbidity and mortality worldwide. Bacterial infections continue to be a major public health concern worldwide, mainly due to the lack of efficacious treatment or the emergence of antibiotic resistance [1], [2]. In particular, a bacterial pathogen that continues to cause significant morbidity is the Gram-negative bacteria *Salmonella,* which has over 2500 serovars and infects a range of hosts including domestic animals, birds, rodents and humans. In humans, *Salmonella enterica* serovar Typhimurium (*Salmonella* Typhimurium) causes diarrheal non-typhoidal salmonellosis (NTS) while *Salmonella* Typhi is the etiologic agent of typhoid fever. The most recent estimates of the global burden of gastroenteritis due to *Salmonella* species is approximately 93.8 million cases with 155,000 deaths annually [3] while the estimated total number of typhoid fever cases globally in 2010 was 26.9 million [4].

Susceptibility to infection is considered to be a complex trait, and significant advances in understanding the host response to bacterial infections have been made using mouse models [5]. As *Salmonella* Typhi is a human-restricted pathogen, the mouse model of the systemic phase of typhoid has been developed using *Salmonella* Typhimurium. Injection of a sub-lethal dose of *Salmonella* Typhimurium via the tail vein results in a systemic infection presenting several clinical and pathological similarities with human typhoid disease. A wide range of susceptibilities to infection with *Salmonella* Typhimurium has been reported among various laboratory mouse strains [6]. The most commonly used inbred strain C57BL/6J is highly susceptible to infection due to a mutation in the gene *Slc11a1* [*7*] *.* Another highly susceptible strain is the wild-derived MOLF/Ei strain despite harboring functional copies of the genes *Slc11a1* and other known *Salmonella*-susceptibility genes [8]. Using an F2 cross between C57BL/6J and MOLF/Ei, we identified three loci affecting susceptibility to *Salmonella* infection in MOLF/Ei mice: *Slc11a1* (*Ity), Ity2 and Ity3* with LOD scores of 18.8, 7.0 and 5.0 respectively. The *Ity2* and *Ity3* loci were only detected in the presence of functional MOLF/Ei *Slc11a1* alleles. In this genetic context, MOLF/Ei alleles at the *Ity2* locus conferred resistance while MOLF/Ei allele at the *Ity3* locus resulted in increased susceptibility to infection [8]. Using congenic mice we have previously confirmed the effect of the *Ity3* locus on *Salmonella* susceptibility and suggested *Ncf2* as a candidate gene underling the *Ity3* locus [9]. *Ncf2* encodes the p67^phox^ component of the NADPH oxidase complex, which is known to be important in controlling *Salmonella* replication [10].

In the current study, we report the generation of a panel of 12 subcongenic strains that have helped refine the *Ity3* locus to a 23.2 Mb interval. A comprehensive phenotypic analysis of these subcongenic strains revealed that the genomic structure of *Ity3* is complex and comprises at least two subloci, *Ity3.1,* and *Ity3.2*. The *Ity3.1* sub-locus corresponds to a decrease in activity of *Ncf2* and is characterized by decreased ROS production, reduced inflammatory cytokine response and increased bacterial burden. The *Ity3.2* sublocus is characterized by a hyper responsive inflammatory cytokine phenotype after exposure to *Salmonella*. These results provide new insights into the genetic architecture of the region of chromosome 1 carrying *Ity3*, a region known to harbour several QTLs linked to inflammatory and autoimmune diseases [11].

## Materials and Methods

### Ethics Statement

All animals were maintained at the Animal Care Facility of McGill University according to the guidelines of the Canadian Council on Animal Care (CCAC). The animal protocol for this study was approved by the McGill University Animal Care Committee (UACC, protocol no. 3285).

### Generation of Subcongenic Lines

**Figure 1:**
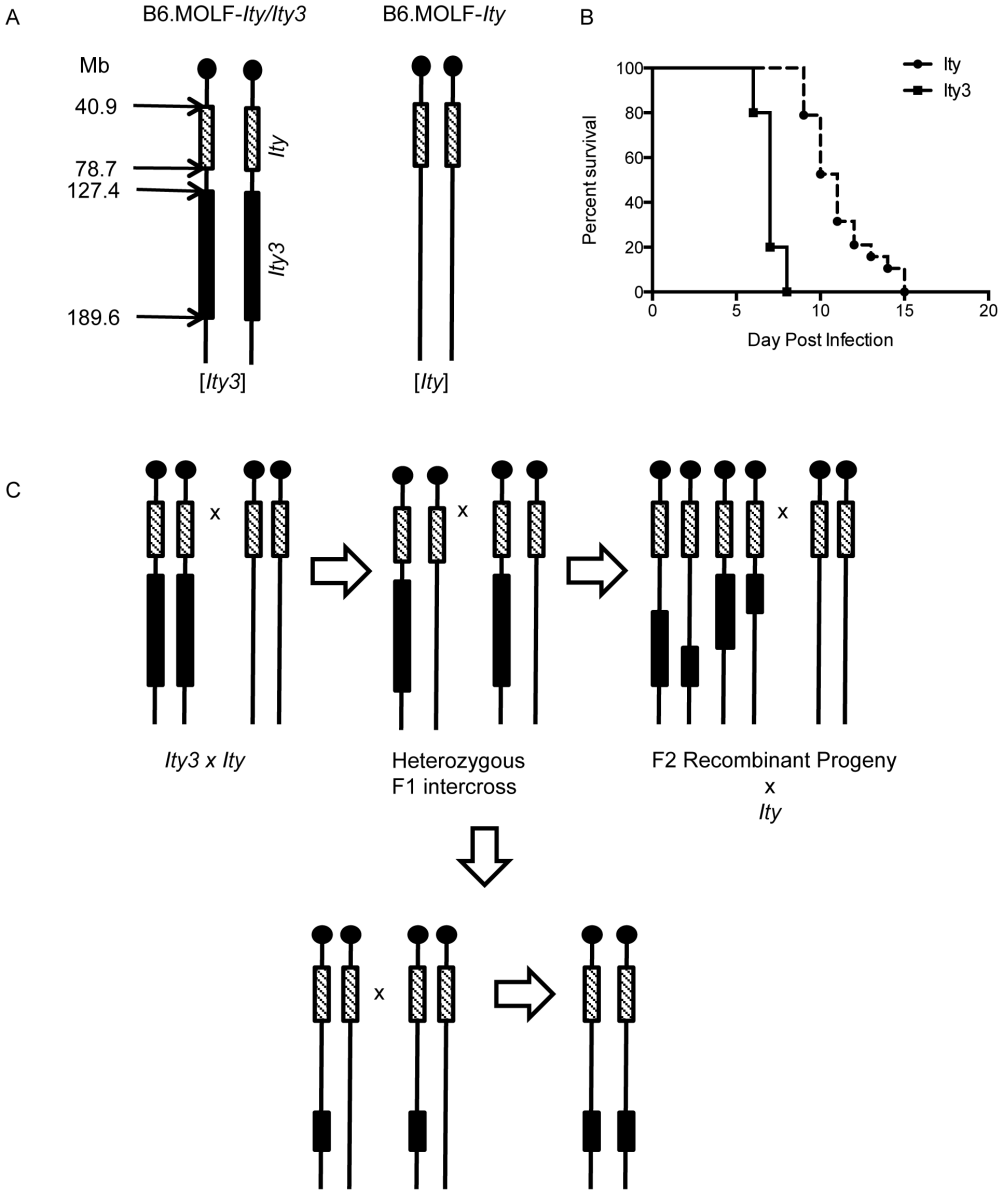
Breeding scheme used to generate the sub-congenic mice. A) Schematic representation of mouse chromosome 1 illustrating the position of the congenic intervals *Ity* and *Ity3* in *B6.MOLF-Ity/Ity3* and *B6.MOLF-Ity* mice that were used to generate the subcongenic strains. The position of the congenic intervals is shown in megabases (Mb) according to Ensembl mouse genome Browser. (B) Survival curves of parental *B6.MOLF-Ity/Ity3 (Ity3)* and *B6.MOLF-Ity (Ity)* mice. *Ity3* congenic mice are more susceptible to *Salmonella* infection, with a Log-Rank (Mantel-Cox) p-value <0.0001. Using the parental *Ity* and *Ity3* congenic strains, subcongenic mice were generated using the breeding scheme shown in (C). The *Ity* and *Ity3* mice were intercrossed to generate heterozygous mice, which were further backcrossed to the parental *Ity* mice. Using marker-assisted breeding, 12 subcongenic strains were generated. Boxes represent the MOLF/Ei allele, while the solid black lines represent the C57BL/6J allele. The shaded boxes represent the MOLF/Ei allele at the *Ity* locus and solid black boxes represent the MOLF/Ei allele at the *Ity3* locus.

The generation of congenic strains B6.MOLF-*Ity (Ity)* and B6.MOLF-*Ity/Ity3 (Ity3)*, which carry the MOLF/Ei allele at the *Slc11a1* gene, have been described previously [12]. A new panel of subcongenic mice for the *Ity3* locus were created by breeding the *Ity* and *Ity3* mice to generate F1 mice heterozygous for the *Ity3* locus (Figure 1a). Recombination within the *Ity3* locus was achieved by inter-crossing the F1 progeny to generate F2 progeny with numerous break points within the *Ity3* locus. Genotyping was performed using microsatellite markers distributed within the *Ity3* locus and 12 breakpoints within the *Ity3* locus were identified. Animals containing the segment of interest were backcrossed with the parental *Ity* stain to generate N2 mice. Using marker-assisted breeding, mice with identical breakpoints were intercrossed to generate homozygous mice. This led to the establishment of twelve recombinant B6.MOLF-*Ity/Ity3* strains that are different from the strains initially described by us [12].

### DNA Extraction and Whole Genome Genotyping

DNA was extracted using a phenol-chloroform extraction, as described previously [13] from tail clippings of all subcongenic strains, as well as C57BL/6J and MOLF/Ei, *Ity* and *Ity3* congenic mice as controls. Whole genome SNP genotyping was done using an Illumina mouse medium density linkage panel that had 1,449 SNPs of which 731 were informative between the C57BL/6J and MOLF/Ei.

### In-vivo Salmonella Infections

Mice were infected with 3000 CFUs of *Salmonella* Typhimurium strain Keller as previously described [9]. Briefly, bacteria were grown on tryptic soy broth to an OD_600_ of 0.1 and cooled to 4°C for an hour. The bacteria were then plated overnight on tryptic soy agar. The following day, the infectious dose was adjusted to 15×10^3^ CFU/ml and 200 ul was injected in the caudal vein of 8–12 week old mice of both sexes. The animals were monitored 2–3 times daily and mice showing signs of distress (piloerection, sunken eyes and abdomen, lethargy) or body condition scoring less than 2.0 [14] were used for clinical endpoints. Survival analysis was conducted using a Kaplan-Meier survival test.

### *In vivo* Bacterial Burden and Serum Cytokines

For bacterial burden quantification in the spleen and the liver, mice were euthanized using CO_2_ and at the required day post infection both organs were removed aseptically, weighed and homogenized using a Polytron (Kinematica, Bohemia, NY). The resulting homogenate was diluted in 0.9% saline and plated on tryptic soy agar to determine organ bacteria burden. Blood was collected by cardiac puncture from uninfected (day 0) mice, as well as at day 3 and day 5 post infection. The sera were collected after centrifugation and frozen at −80°C. Serum cytokine levels for IL-6, IFN-γ, TNF, IL-12 were measured using sandwich ELISAs (eBioscience) following the manufacturer’s protocol.

### Cells Preparation and PAMP Stimulation

Spleens were aseptically removed from uninfected mice and transferred to growth medium (RPMI 1640). Each spleen was briefly ground between the frosted areas of two sterile microscope slides to generate a cell suspension. This suspension was passaged through a 21 and 25-gauge needle to separate any cellular aggregates. The cells were centrifuged at 1500 r.p.m. for 5 minutes and re-suspended in red blood cells lysis solution (Invitrogen, Burlington, Ontario, Canada) for 5 minutes. The cells were washed and re-suspended in RPMI 1640 supplemented with 10% FBS (HyClone), 100 U/ml penicillin G and 100 µg/ml streptomycin sulfate (Life Technologies). Splenocytes were stimulated overnight with 1 mM CpG DNA (Alpha DNA), 10 mg/ml zymosan (no. TLRL-ZYN; InvivoGen, San Diego, CA), 10 mg/ml LPS055:B5 (no. L6529; Sigma-Aldrich), 10 mg/ml lipoteichoic acid (no. L2515; Sigma-Aldrich), or RPMI alone to measure IL-6 using sandwich ELISAs.

Bone marrow derived macrophages (BMDM) were isolated from femurs of 10–16 week-old male mice. The cells were washed and re-suspended in 10 ml growth medium (RPMI 1640), containing 10% FBS (HyClone), 100 U/ml penicillin G and 100 µg/ml streptomycin sulfate (Life Technologies). The cell suspension was plated on non-adherent bacteriological-grade Petri dishes (Fisher) and supplemented with 30% (vol/vol) L929 cell-conditioned medium as a source of M-CSF and maintained in culture for 5 days. BMDMs were harvested and plated on 6-well plates at a density of 10^6^ cells/ml and cultured for an additional 16–24 hours.

### *In-vitro Salmonella Typhimurium* Infection, Nitric Oxide and Cytotoxicity Assessment

BMDMs were primed with 20 ng/ml IFN-γ overnight prior infection. *Salmonella* Typhimurium was added to the cells at multiplicities of infection of 10∶1. After a period of 45 minutes, RPMI 1640 medium containing 100 µg/ml gentamycin was added for a period of 1 hour. The medium was replaced by RPMI 1640 containing 10 µg/ml (time point 0). Supernatants were collected for LDH, NO and cytokine measurement at 2, 4 and 6 hours following the addition of a 10 µg/ml gentamycin. Cells were lysed with with 1% Triton-X 100 diluted in PBS and bacterial counts were determined by serial dilutions of the lysates on trypticase soy agar. Nitric oxide production was estimated by measuring the accumulation of nitrate using the Griess reaction (Promega) and lactate dehydrogenase (LDH) released from cells was measured as per manufacturer’s (Roche) instructions.

### Reactive Oxygen Species Production

Bone marrow derived cells were plated on non-tissue culture treated petris and incubated with LPS or heat killed *Salmonella* at an MOI of 1∶20 for 6 hours. RPMI was aspirated and the cells were washed with warm PBS and incubated with CM-H_2_DCFDA (10 µM, 30 min; Invitrogen) in serum-free medium. Cells were washed with warm PBS, then removed from the well using cold PBS containing 10 mM EDTA, pelleted at 1200 r.p.m., resuspended in cold PBS containing 1% FBS and analysed using a FACSCanto (BD Biosciences) with FlowJo software (Tree Star). Mean fluorescence intensity were used as an indicator for the ROS production.

### Statistical Analysis

Statistical analysis was performed using Graph Pad Prism 6 (GraphPad Software, San Diego, CA).

## Results

### Refinement of the Ity3 Interval Using Sub-congenic Mice

We have shown previously that the transfer of *Ity* and *Ity3* from MOLF/Ei on a C57BL/6J background (B6.MOLF-*Ity* and B6.MOLF-*Ity/Ity3*) clearly impact on susceptibility to infection [12]. At that time B6.MOLF-*Ity/Ity3* ^MOLF/B6^ congenic mice were used as controls because B6.MOLF-*Ity* (*Ity3^B6/B6^*) mice were unavailable. B6.MOLF-*Ity/Ity3* mice were significantly more susceptible to infection (mean survival time (MST) = 8.4±0.3 days) compared to those that carry only one MOLF/Ei allele at *Ity3*, B6.MOLF-*Ity/Ity3* ^MOLF/B6^ (MST = 10.6±0.9 days). In the current paper, we used mice that were homozygous at both loci (Figure 1A). MST was 11.0±2.5 days in B6.MOLF-*Ity* compared to 7.0±1.8 days in B6.MOLF-*Ity/Ity3* (Figure 1B) suggesting some co-dominant effect of MOLF/Ei and C57BL/6J alleles at *Ity3* on survival to infection.

**Figure 2:**
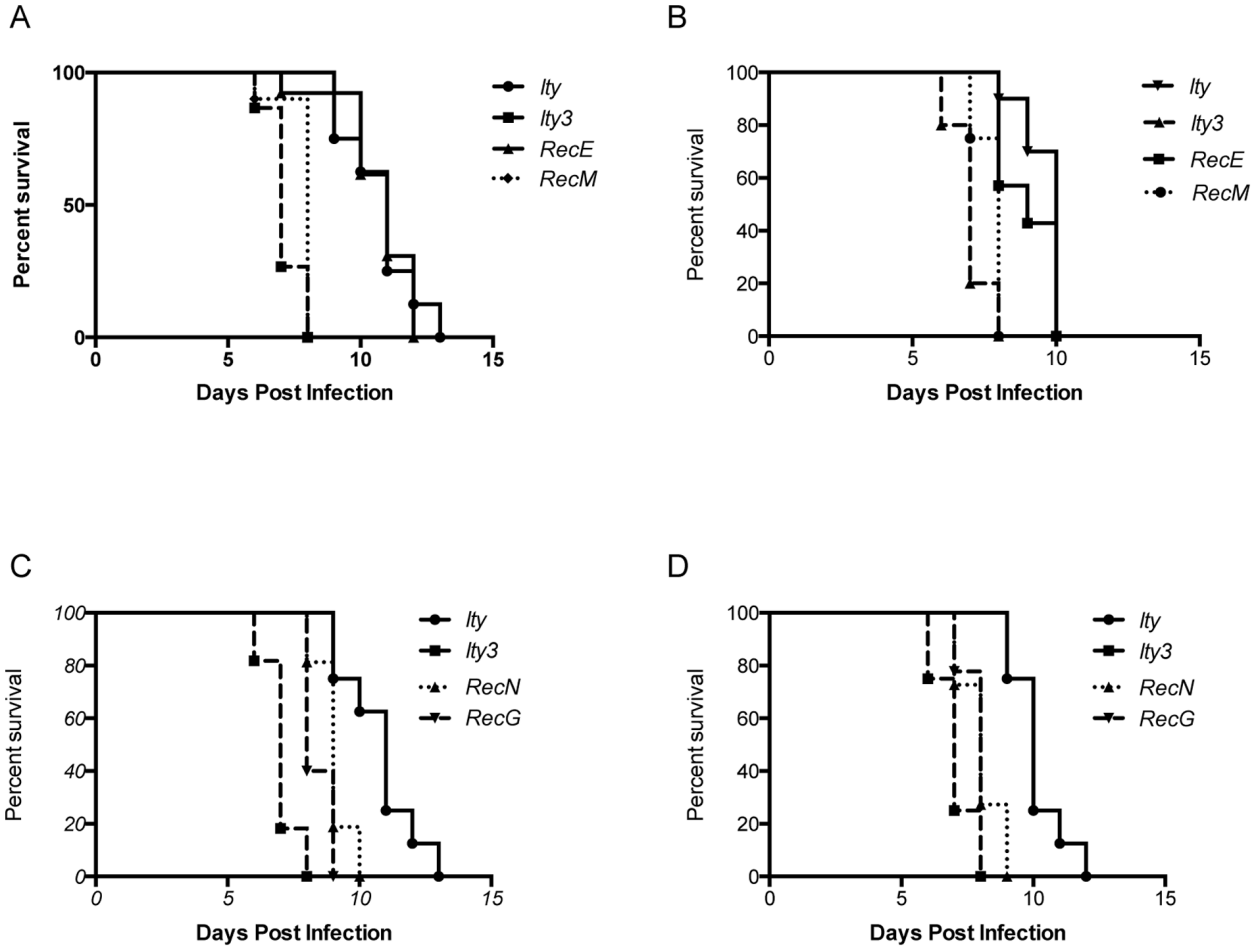
Survival curves of male and female congenic and sub-congenic mice after infection with *Salmonella* Typhimurium. The left panel (A) and (C) show the survival of male mice after IV infection with *Salmonella* Typhimurium while the right panel (B) and (D) show the survival of female mice after infection. Based on the survival curves and mean survival time, strains were classified as either *Ity-like* or *Ity3-like* (A, B) Two strains, *Ity3.*Rec*N* and *Ity3.*Rec*G* were identified as having an intermediate phenotype (C, D). Log-Rank (Mantel-Cox) for males: p = 0.0025 *Ity3.RecG* versus *Ity*; p = 0.0035 *Ity3.RecG* versus *Ity3*; p = 0.0008 *Ity3.RecN* versus *Ity*; p<0.0001 *Ity3.RecN* versus *Ity3*. Log-Rank (Mantel-Cox) for females: p = 0.0001 *Ity3.RecG* versus *Ity*; p = 0.0520 *Ity3.RecG* versus *Ity3*; p = 0.0001 *Ity3.RecN* versus *Ity*; p = 0.0461 *Ity3.RecN* versus *Ity3*.

In order to further refine the 62.2 Mb *Ity3* locus, we generated 12 subcongenic strains (*Ity3.RecA* to *Ity3.RecS*) using marker assisted backcrossing (Figure 1). We used a medium density genotyping array to genotype all 12 subcongenic mice to ensure that any phenotypic differences observed between the sub-congenic strains was due solely to the *Ity3* sub-congenic segments. Using this approach no MOLF/Ei alleles were detected outside the *Ity* and *Ity3* regions. This experiment also allowed for a more precise mapping of the boundaries of the different recombinant *Ity3* segments (Table 1). All 12 subcongenic strains were evaluated for susceptibility to *Salmonella* infection. Representative survival analyses are presented in Figure 2. The analysis was done separately in males and females. The difference in survival was more significant in males as compared to females as shown in Figure 2. The male *Ity* strain was more resistant to infection than female *Ity* mice. In male mice, the survival curve of recombinant strain *Ity3.RecM* was undistinguishable from that of *Ity3* and strain *Ity3.RecE* presented a phenotype similar to the *Ity* control (Figure 2A). A similar observation was made for *Ity3.RecM* and *Ity3.RecE* in females (Figure 2B). Based on the survival curves and the mean survival time post infection, the sub-congenic strains were classified as either *Ity*-like or *Ity3*-like (Table 1). We also identified two sub-congenic strains, *Ity3.RecG* and *Ity3.RecN* that showed an intermediate survival phenotype (Figure 2C–D). These strains carry complementary segments of the *Ity3* interval and independently lead to an intermediate phenotype with MST of 8.6±1.7 days and 8.0±2.1 days in *Ity3.RecN* and *Ity3.RecG* sub-congenic strains respectively. Based on the survival analyses of the 12 subcongenic mice, the *Ity3* interval was refined to a ∼23 Mb interval on chromosome 1 located between position 151.0 Mb and 174.2 Mb (Ensembl build 37). The identification of the two subcongenic strains presenting an intermediate phenotype is suggestive of the presence of at least two loci within the *Ity3* locus: *Ity3.1* sub-locus located between 151 Mb and 156.2 Mb and *Ity3.2* from 158.6 Mb to 174.2 Mb, that together contribute to the enhanced susceptibility of the *Ity3* congenic mice (Table 1).

**Table 1:** Fine mapping of the *Ity3* interval using 12 sub-congenic strains.

Table 1:															
SNP's	Position	Ity	Ity3	B	C	D	E	F	G	I	S	A	M	N	P
D1Mit218	127.4	B6	M	M	M	M	M	M	M	M	B6	B6	B6	B6	B6
rs3724826	131.5	B6	M	B6	M	M	M	M	M	M	B6	B6	B6	B6	B6
D1Mit193	131.2	B6	M	B6	M	M	M	M	M	M	B6	B6	B6	B6	B6
Chi3l1	134.1	B6	M	B6	B6	M	M	M	M	M	M	B6	B6	B6	B6
rs6279930	137.3	B6	M	B6	B6	B6	M	M	M	M	M	B6	B6	B6	B6
D1Mit197	136.3	B6	M	B6	B6	B6	M	M	M	M	M	B6	B6	B6	B6
rs13476141	140.5	B6	M	B6	B6	B6	B6	M	M	M	M	B6	B6	B6	B6
D1Mit448	141.1	B6	M	B6	B6	B6	B6	M	M	M	M	B6	B6	B6	B6
rs6382880	142.5	B6	M	B6	B6	B6	B6	M	M	M	M	B6	B6	B6	B6
rs13476163	146.8	B6	M	B6	B6	B6	B6	M	M	M	M	M	B6	B6	B6
D1Mit194	147.0	B6	M	B6	B6	B6	B6	M	M	M	M	M	B6	B6	B6
D1Mit102	147.2	B6	M	B6	B6	B6	B6	M	M	M	M	M	B6	B6	B6
D1Mit288	149.3	B6	M	B6	B6	B6	B6	M	M	M	M	M	M	B6	B6
rs6393307	151.0	B6	M	B6	B6	B6	B6	M	M	M	M	M	M	B6	B6
***Ncf2***	**152.8**	**B6**	**M**	**B6**	**B6**	**B6**	**B6**	**B6**	**M**	**M**	**M**	**M**	**M**	**B6**	**B6**
**D1Mit14**	**156.8**	**B6**	**M**	**B6**	**B6**	**B6**	**B6**	**B6**	**M**	**M**	**M**	**M**	**M**	**M**	**B6**
**rs3719034**	**157.2**	**B6**	**M**	**B6**	**B6**	**B6**	**B6**	**B6**	**M**	**M**	**M**	**M**	**M**	**M**	**B6**
**rs6387609**	**157.9**	**B6**	**M**	**B6**	**B6**	**B6**	**B6**	**B6**	**M**	**M**	**M**	**M**	**M**	**M**	**B6**
**rs3693161**	**160.3**	**B6**	**M**	**B6**	**B6**	**B6**	**B6**	**B6**	**B6**	**M**	**M**	**M**	**M**	**M**	**B6**
***Fasl***	**161.8**	**B6**	**M**	**B6**	**B6**	**B6**	**B6**	**B6**	**B6**	**M**	**M**	**M**	**M**	**M**	**B6**
**rs13476208**	**162.0**	**B6**	**M**	**B6**	**B6**	**B6**	**B6**	**B6**	**B6**	**M**	**M**	**M**	**M**	**M**	**B6**
***Sell***	**164.1**	**B6**	**M**	**B6**	**B6**	**B6**	**B6**	**B6**	**B6**	**M**	**M**	**M**	**M**	**M**	**B6**
***Selp***	**164.1**	**B6**	**M**	**B6**	**B6**	**B6**	**B6**	**B6**	**B6**	**M**	**M**	**M**	**M**	**M**	**B6**
**D1Mit63**	**165.3**	**B6**	**M**	**B6**	**B6**	**B6**	**B6**	**B6**	**B6**	**M**	**M**	**M**	**M**	**M**	**B6**
**rs13476219**	**166.2**	**B6**	**M**	**B6**	**B6**	**B6**	**B6**	**B6**	**B6**	**M**	**M**	**M**	**M**	**M**	**B6**
D1Mit403	175.6	B6	M	B6	B6	B6	B6	B6	B6	B6	M	M	M	M	B6
rs13476273	182.1	B6	M	B6	B6	B6	B6	B6	B6	B6	M	M	M	M	B6
rs3667164	188.7	B6	M	B6	B6	B6	B6	B6	B6	B6	M	B6	M	M	M
D1Mit17	189.6	B6	M	B6	B6	B6	B6	B6	B6	B6	M	B6	M	M	M
Survival				Ity	Ity	Ity	Ity	Ity	INT	Ity3	**Ity3**	**Ity3**	**Ity3**	INT	Ity
n =		82	39	32	20	11	23	64	45	30	21	36	65	65	14

a Position (Mb) based on Ensembl Build 37;

b B6 corresponds to the C57BL/6J allele;

c M corresponds to the MOLF/Ei allele;

d n represents the number of mice tested;

e the sub-congenic strains nomenclature is shortened showing only the letter attributed to each strain.

f The survival was classified, as either *Ity*-like (Ity) or *Ity3*-like (Ity3) or intermediate (INT).

### Contribution of Ity3 to High Bacterial Burden in Congenic and Subcongenic Mice

To characterize the phenotypic effect of *Ity3* and *Ity3.1* and *Ity3.2* sub-loci on susceptibility to *Salmonella* infection, we carried out subsequent analyses using *Ity*, *Ity3*, *Ity3.RecG* and *Ity3.RecN* strains. These mice were evaluated for a number of additional phenotypes known to be related to *Salmonella* susceptibility including bacterial load in target tissues, clinical hematology, and pathology. As previously observed in other strains of mice, all four strains developed a mild anemia, lymphopenia and neutrophilia during infection (data not shown). No significant differences were seen between the strains in either infected or uninfected mice. Baseline and post-infection histology (H&E staining) of target organs (spleen and liver) showed no obvious histopathological difference between strains (data not shown).

**Figure 3:**
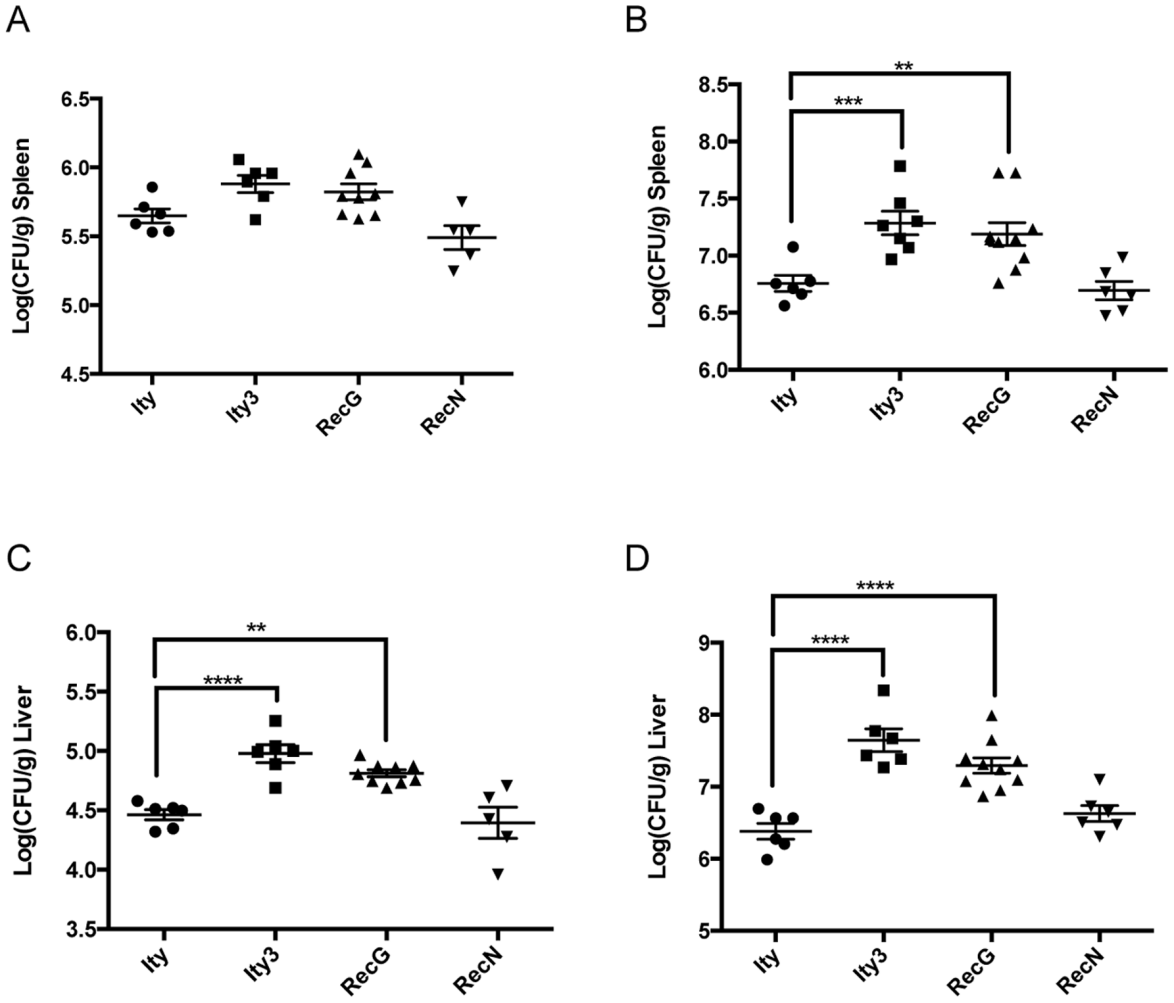
Bacterial load in the spleen and liver of male congenic and subcongenic strains at day 3 and day 5 post-infection with *Salmonella* Typhimurium. The bacterial burden in the spleen (A, B) and liver (C, D), is shown at day 3 (A, C) and day 5 (B, D). Significant differences in log CFU of congenic and subcongenic strains are shown with respect to the reference *Ity* strain. At day 3-post infection, there was a significant higher bacterial burden in the liver of the *Ity3* and *Ity3.RecG* strains as compared to the *Ity* strain. At day 5 post infection, the bacterial loads in both the liver and spleen were significantly higher in *Ity3.RecG* and *Ity3* males. The data were analysed by one-way ANOVA followed by Dunnett’s multiple-comparisons testing. *corresponds to a P-value of ≤0.05, **corresponds to a P-value of ≤0.01, ***corresponds to P-value ≤0.001, while ****implies a P≤0.0001.

**Figure 4:**
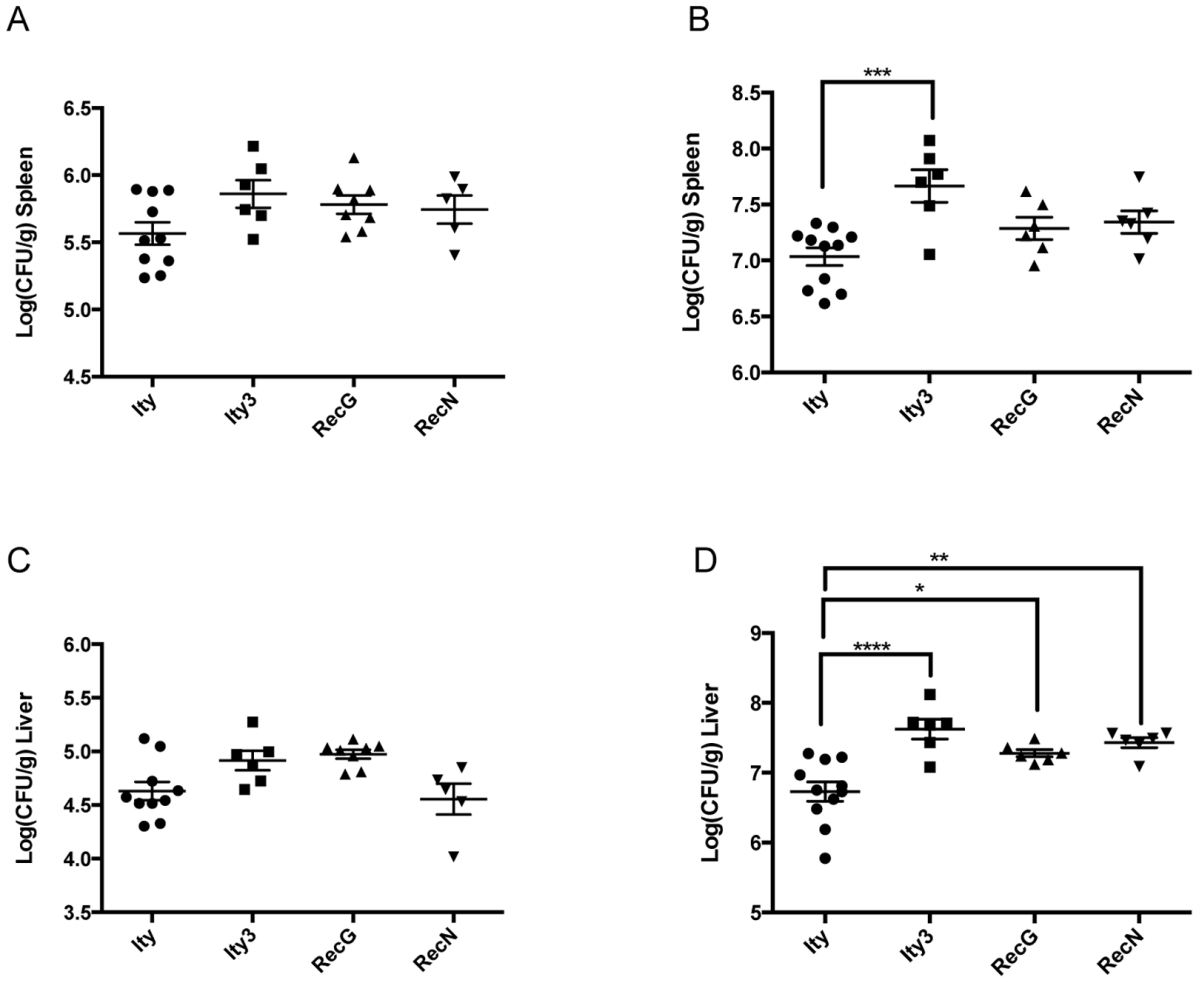
Bacterial load in the spleen and liver of female congenic and subcongenic strains at day 3 and day 5 post-infection with *Salmonella* Typhimurium. The bacterial burden in the spleen (A, B) and liver (C, D), is shown at day 3 (A, C) and day 5 (B, D). Significant differences in log CFU of congenic and subcongenic strains are shown with respect to the reference *Ity* strain. At day 5-post infection, the bacterial loads in both the liver and spleen were significantly higher in *Ity3* females compared to *Ity* mice. The subcongenic strains *Ity3.RecG* and *Ity3.RecN* presented an intermediate phenotype. The data were analysed by one-way ANOVA followed by Dunnett’s multiple-comparisons testing. *corresponds to a P-value of ≤0.05, **corresponds to a P-value of ≤0.01, ***corresponds to P-value ≤0.001, while ****implies a P≤0.0001.

To determine if MOLF/Ei allele at *Ity3* results in increase bacterial proliferation, we measured tissue bacterial burden after challenged with *Salmonella* Typhimurium in the spleen and liver of congenic and subcongenic mice on day 3 and day 5 post-infection. At day 3 post-infection no major differences were seen in the spleen of either males or females (Figure 3A and 4A). Higher bacterial burden was detected only in the liver of males from *Ity3* and *Ity3.RecG* strains compared to *Ity* mice (Figure 3C and 4C). More pronounced differences were seen in both sexes at day 5 post infection. Significant higher bacterial load was detected in both the spleen and liver of *Ity3* male and female mice when compared to the *Ity* strain (Figure 3B, 3D, 4B, 4D). In males, the two subcongenic mice presenting an intermediate survival phenotype behaved differently with respect to bacterial load, *Ity3.RecG* presented high bacterial load similarly to *Ity3* and *Ity3.RecN* showed bacterial load similar to those observed in *Ity* mice (Figure 3B, 3D). In female mice, *Ity3.RecG* and *Ity3.RecN* strains showed intermediate phenotypes (Figure 4B and 4D). These data showed that the MOLF/Ei *Ity3* locus contributed to high bacterial load in the spleen and liver of male and female congenic mice and suggest the involvement of the *Ity3.1* locus.

### Impact of Ity3 on the Inflammatory Response

**Figure 5:**
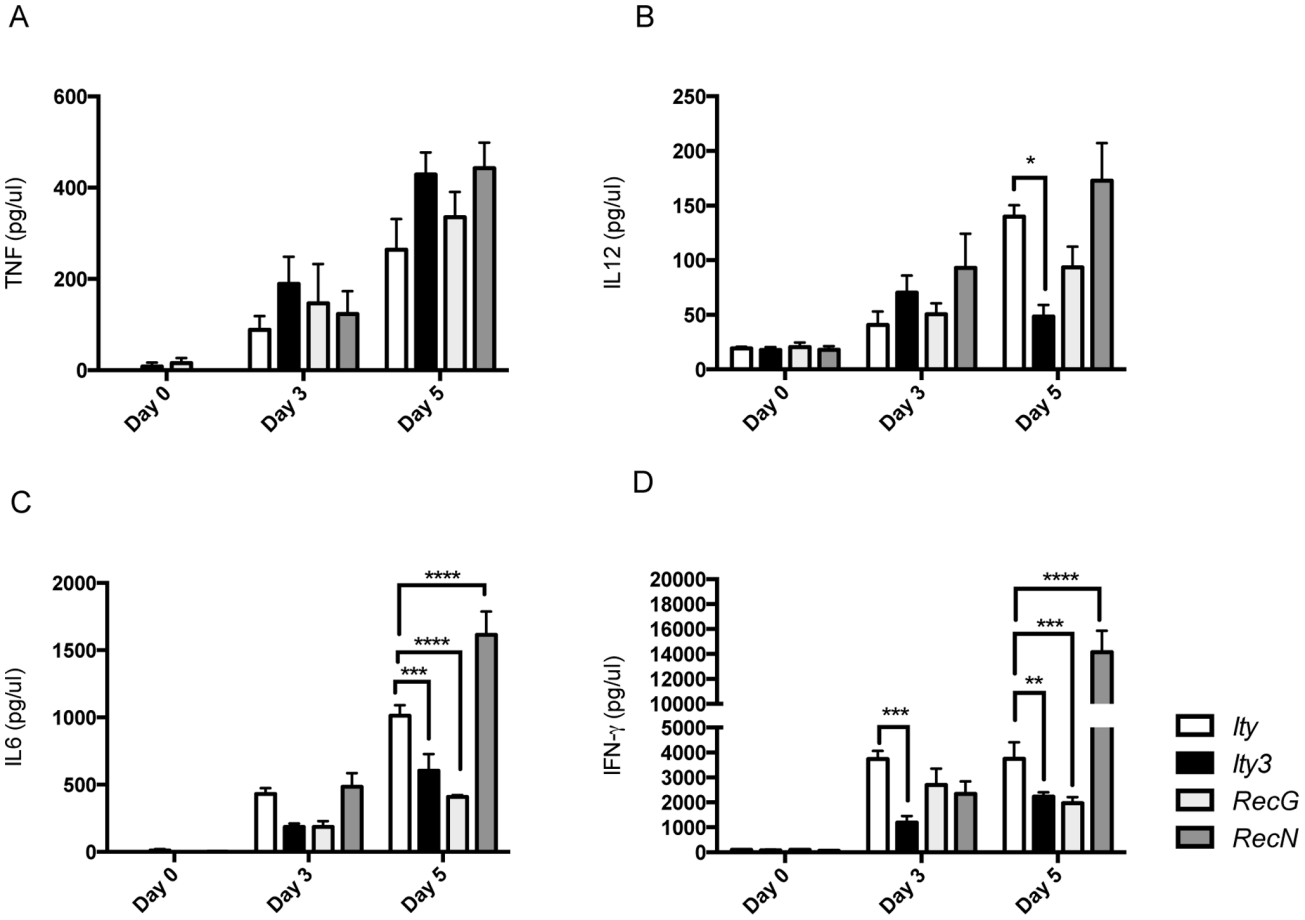
Serum cytokine levels in *Ity*, *Ity3, Ity3.RecN* and *Ity3.RecG* male mice during infection with *Salmonella* Typhimurium. TNF (A), IL-12 (B), IL-6 (C) and IFN-γ (D) were measured in the serum of congenic *Ity* (n = 4) and *Ity3* (n = 4) and sub-congenic *Ity3.RecN* (n = 4) and *Ity3.RecG* (n = 4) mice before infection and at day 3 and day 5 post infection. The data shown are representative of two experiments with n = 4 per experiment. All levels were compared to the resistant strain *Ity* at each time point. No major differences were observed in the expression of TNF across the four strains (A). The data were analysed by two-way ANOVA followed by Dunnett’s multiple-comparisons testing. *corresponds to a P-value of ≤0.05, **corresponds to a P-value of ≤0.01, ***corresponds to P-value ≤0.001, while ****implies a P≤0.0001.

To further dissect the mechanism involved in increased bacterial load in *Ity3* mice, we examine the impact of *Ity3.1* and *Ity3.2* on the inflammatory response to infection, by evaluating cytokine production during infection *in vivo* (in the sera), *ex vivo* (splenocytes) and during *in vitro* infection of BMDM from *Ity*, *Ity3*, *Ity3.RecG* and *Ity3.RecN* mice. Early during *Salmonella* infection, immune cells including macrophages, granulocytes and lymphocytes are central to control bacterial replication by producing IL-6, TNF, IFN-γ and IL-12 [15], [16]. We then measured these four cytokines in the sera of male and female mice at day 0 and at day 3 and 5 post infection. All four cytokines were found in greater amount in the sera of males and females during infection. Similar results were observed in both males and females although significant differences in serum cytokine levels were detected only in male congenic and subcongenic strains (Figure 5). There was no variation among strains for TNF production (Figure 5A). Similar differences between *Ity* and *Ity3* were observed for IL-6, IFN-γ and IL-12 at day 5 post-infection with *Ity3* presenting significant lower levels of these three cytokines when compared to *Ity* (Figure 5B–D). The strain *Ity3.RecG* also presented low levels of IL-6 and IFN-γ when compared to the levels detected in *Ity* (Figure 5C–D) whereas strain *Ity3.RecN* presented cytokine levels that were either equivalent to those observed in *Ity* (Figure 5B) or significantly higher (Figure 5C–D). Even though the two loci have opposite effects on the production of cytokines, the overall production of IL6, IFN-γ and IL-12 in response to *Salmonella* infection *in vivo* by *Ity3* congenic was significantly lower compared to the *Ity* strain suggesting the existence of interaction between *Ity3.1* and *Ity3.2*.

**Figure 6:**
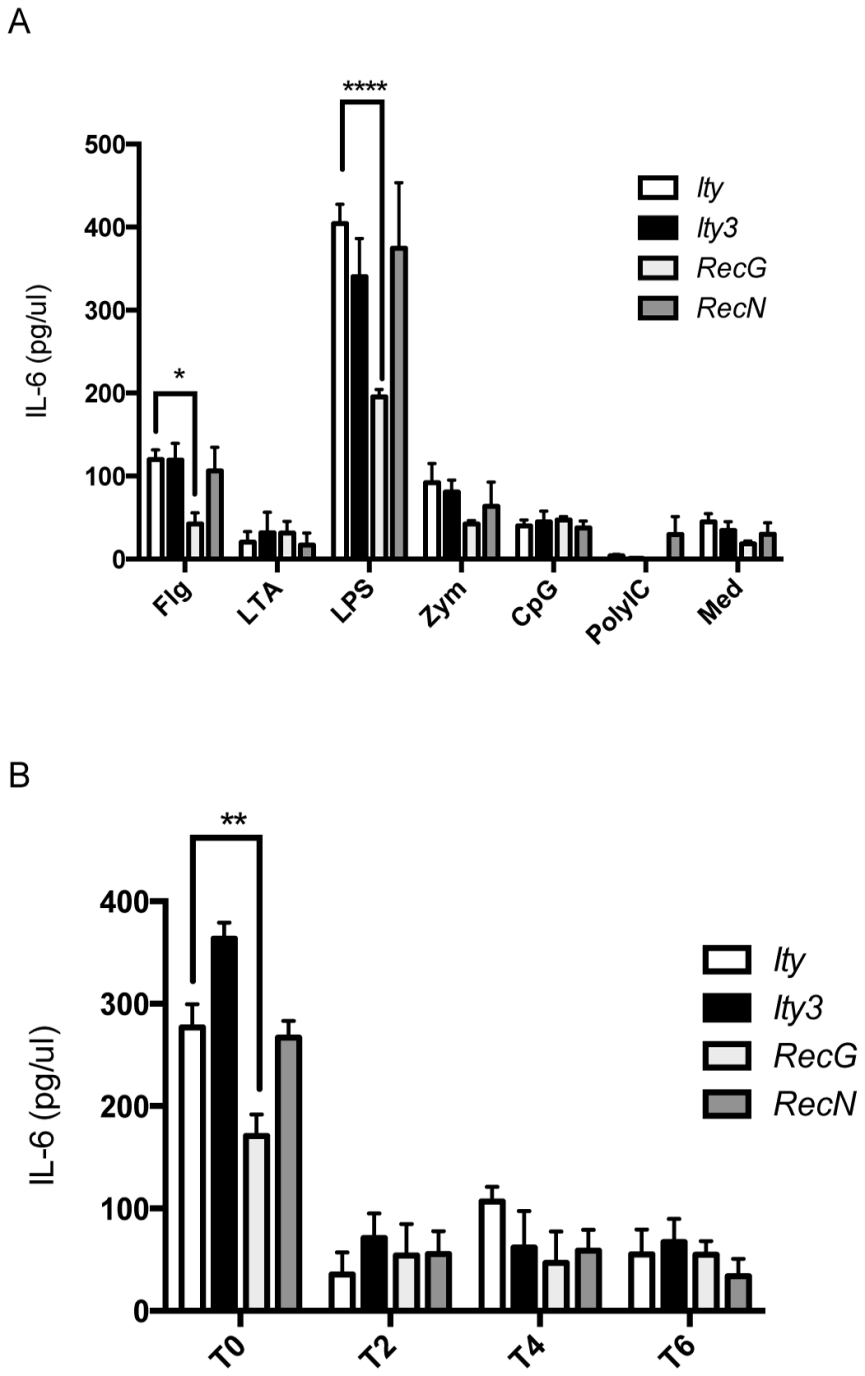
IL-6 production by explanted splenocytes and BMDM from *Ity*, *Ity3, Ity3.RecN* and *Ity3.RecG* after stimulation with PAMPs or infection with *Salmonella* Typhimurium. A) IL-6 production by explanted splenocytes after stimulation with flagellin (Flg), lipoteichoic acid (LTA), lipopolysaccharide (LPS), zymosan (Zym), CpG-DNA (CpG), and PolyIC. The culture medium (Med) was used as negative control. *Ity* (n = 4), *Ity3* (n = 4), *Ity3.RecN* (n = 4) and *Ity3.RecG* (n = 4). Significant differences in IL-6 production are shown with respect to the reference *Ity* strain. Splenocytes from *Ity3.RecG* strain were hypo-responsive to stimulation with *Tlr4* and *Tlr5* ligands as shown by significant lower levels of IL-6 after stimulation with LPS and flagellin, respectively, as compared to the *Ity* strain. No major differences were seen in IL-6 production in *Ity3* and *Ity3.RecN* strains implying that these strains do not have a defect in TLR-dependent activation of IL-6 production. The data shown are representative of two experiments with n = 4 per experiment B) IL-6 production measured in the supernatant of BMDM derived from *Ity* (n = 4), *Ity3* (n = 4), *Ity3.RecN* (n = 4) and *Ity3.RecG* (n = 4) male mice. The data shown are representative of three experiments with n = 4 per experiment. BMDM were infected with *Salmonella* Typhimurium. After a period of 45 min, RPMI 1640 containing 100 µg/ml gentamycin was added for a period of 1 h (T0). The medium was subsequently replaced by RPMI 1640 containing 10 µg/ml gentamycin and IL-6 was measured after 2 (T2), 4 (T4) and 6 (T6) hrs of incubation. Significant differences in IL-6 production are shown with respect to the reference *Ity* strain. The data were analysed by two-way ANOVA followed by multiple-comparisons testing. *corresponds to a P-value of ≤0.05, **corresponds to a P-value of ≤0.01, ***corresponds to P-value ≤0.001, while ****implies a P≤0.0001.

To further study cytokine production by strain *Ity3.RecG* and *Ity3.RecN*, explanted splenocytes were stimulated with a number of PAMPs and IL-6 production was measured (Figure 6A). Consistent with the cytokine levels detected in the serum during infection, splenocytes from *Ity3.RecG* presented significant lower levels of IL-6 after stimulation with LPS and flagellin. *In vitro* infection of BMDM also showed a hyporesponsive IL-6 phenotype in *Ity3.RecG* mice (Figure 6B). The hyperresponsive phenotype of *Ity3.RecN* could not be detected *in vitro*. These data suggest that the *Ity3.1* locus is mostly responsible for the observed reduced cytokine production in response to TLR activation.

### Ity3.1 Influences the Production of Reactive Oxygen Species (ROS)

**Figure 7:**
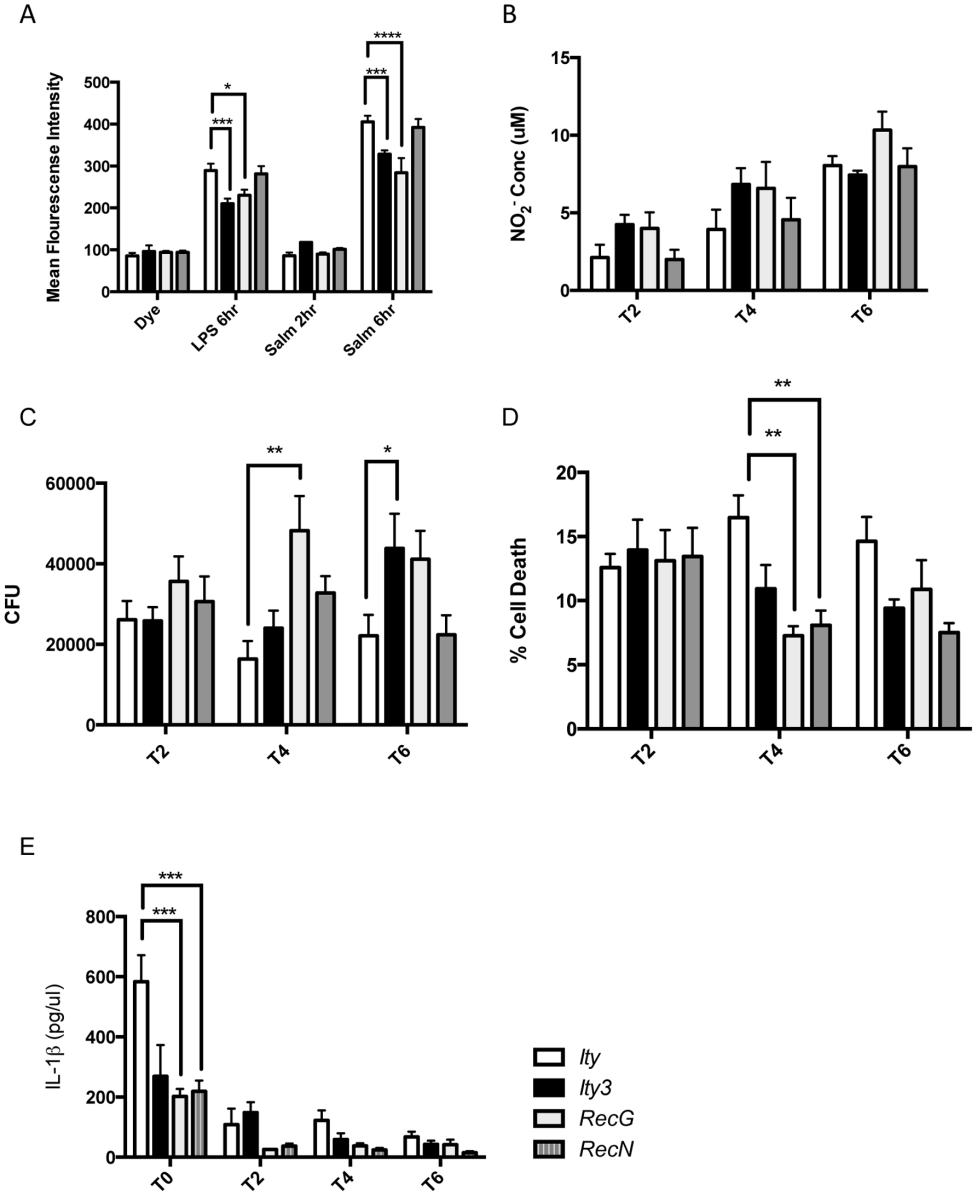
In vitro *Salmonella* Typhimurium infection, reactive oxygen species production, and nitric oxide and cytotoxicity assessment in BMDM derived from *Ity*, *Ity3, Ity3.RecN* and *Ity3.RecG.* A) ROS production by BMDM was quantified following stimulation with either LPS or heat- killed *Salmonella* Typhimurium. Both LPS and a 6-hour incubation with *Salmonella* resulted in reduced H_2_O_2_ production by *Ity3* and *Ity3.RecG* mice. Levels of nitric oxide production following infection with live *Salmonella* Typhimurium (B) were similar in all four strains of mice. C) The bacterial loads per well in *Ity*, *Ity3, Ity3.RecN* and *Ity3.RecG* after infection with *Salmonella* Typhimurium are shown. Bacterial loads were higher in *Ity3* mice (T6) and in *Ity3.RecG* (T4) showing the involvement of the sub-locus *Ity3.1* in controlling bacterial load in vitro. D) LDH assay was used to measure cell death after infection of BMDM with *Salmonella* Typhimurium. Both *Ity3.RecG* and *Ity3.RecN* showed decreased LDH release at T4 suggesting these strains have reduced cell death as compared to the congenic parental *Ity* strains. E) IL-1β was measured in the supernatant of BMDM infected with *Salmonella* and both *Ity3.RecG* and *Ity3.RecN* show reduced IL-1β production. The definition of T0, T2, T4 and T6 is given in the legend of Figure 6. *Ity* (n = 4), *Ity3* (n = 4), *Ity3.RecN* (n = 4) and *Ity3.RecG* (n = 4). The data shown are representative of three experiments with n = 4 per experiment. The data were analysed by two-way ANOVA followed by multiple-comparisons testing. *corresponds to a P-value of ≤0.05, **corresponds to a P-value of ≤0.01, ***corresponds to P-value ≤0.001, while ****implies a P≤0.0001.

In previous studies, we have reported the candidacy of *Ncf2* (a regulatory subunit of the NADPH oxidase) as the gene underlying *Ity3* based on its physical location, expression analysis, coding sequence polymorphism and functional studies [9]. NADPH oxidase is known to play a crucial role in innate immunity by generating ROS that have microbicidal activity and as an activator of many signalling pathways [17]. In the current study, we measured H_2_O_2_ production by BMDM during exposure to LPS and to heat-killed *Salmonella*. Consistent with previous results, *Ity3* showed lower ROS production after LPS and heat-killed *Salmonella* stimulation when compared to the control *Ity* strain (Figure 7A). This effect could be mapped to the *Ity3.1* sublocus, the region harbouring *Ncf2*, since *Ity3.RecG* but not *Ity3.RecN* mice showed reduced production of ROS (Figure 7A). We also assayed nitric oxide production by BMDM derived from *Ity, Ity3, Ity3.RecG* and *Ity3.RecN* strains at various time points post infection, but no significant differences were observed across the various strains (Figure 7B). In order to assess if lower ROS production played a role in bacterial killing in *Ity3.RecG*, we infected BMDM with *Salmonella* and measured bacterial load over time. Significant differences in bacterial count were seen in the strains carrying the MOLF/Ei allele at *Ity3.1* (*Ity3* and *Ity3.RegG*) compared to strains carrying the C57BL/6 allele at *Ity3.1* (*Ity* and *Ity3.RecN*) (Figure 7C). The kinetics of infection were different between *Ity3* and *Ity3.RegG* BMDM with high CFU counts observed at earlier time point in *Ity3.RecG* BMDM. This illustrates that the MOLF/Ei segment at *Ity3.1* leads to reduced bacterial killing by the macrophages, supporting a role for *Ncf2* in susceptibility of MOLF/Ei mice to infection.

Because of the known role of ROS in cell death, we evaluated cell death in BMDM after infection with *Salmonella* by measuring LDH release. Significant lower LDH levels were detected in the two subcongenic *Ity3.RecG* and *Ity3.RecN* mice (Figure 7D). These observations may suggest the presence of an additional MOLF/Ei locus common to *Ity3.RecG* and *Ity3.RecN* that could play a role in cell death during infection. This locus appeared also to decrease the production of the proinflammatory cytokine IL-1β after *in vitro* infection (Figure 7E). Taken together, these data suggest that lower ROS production is paralleled by decreased cell death of primary BMDM infected *in vitro* in *Ity3* and *Ity3.RecG* mice but not in *Ity3.RecN* suggesting that this cellular phenotype is not linked to the *Ity3.1* but rather to an adjacent distal region of *Ity3*.

## Discussion

**Figure 8:**
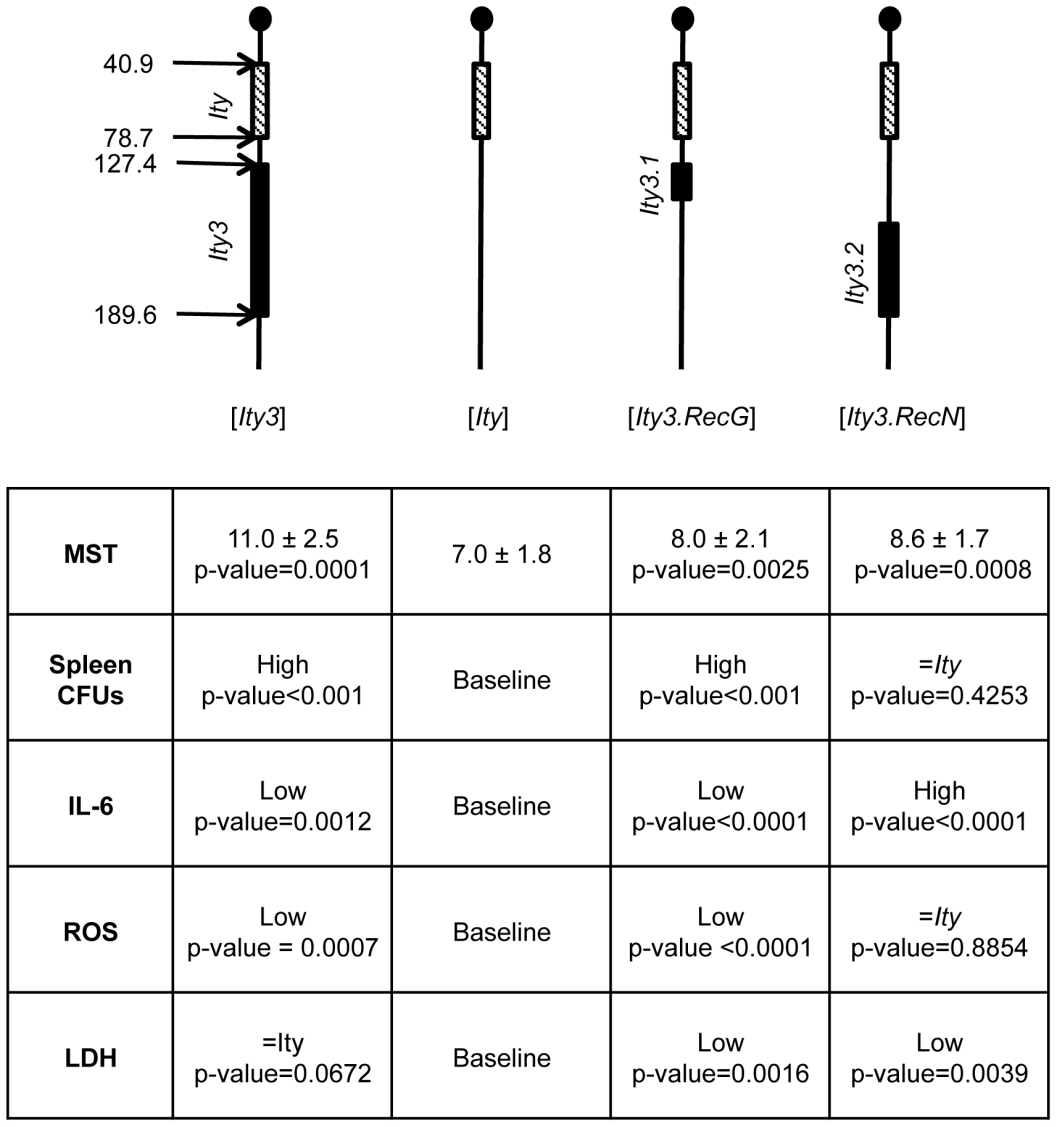
Schematic representation of *Ity3.RegG* and *Ity3.RecN* subcongenic strains. Boxes represent MOLF/Ei-derived congenic intervals. The positions of *Ity3.1* (151.0–156.2 Mb) and *Ity3.2* (158.6–174.2 Mb) are shown on mouse chromosome 1. A summary of selected subphenotypes is shown below the map. Spleen CFUs and IL-6 in the sera were measured 5 days post infection and ROS production and LDH release 6 hrs after exposure of BMDM to *Salmonella*.

In this study, we report the refinement of the *Ity3* locus to a ∼23 Mb interval using a panel of 12 subcongenic strains. We have showed that susceptible *Ity3* mice carried high bacterial load in target organs, and reduced oxidative burst activity and systemic proinflammatory response. We have used the new subcongenic strains to map these phenotypes within the *Ity3* interval and we identified two subloci (*Ity3.1* and *Ity3.2*) that act together to confer susceptibility to infection in the congenic strain *Ity3* (Figure 8). The *Ity3.1* locus was shown to be responsible for high bacterial burden and low ROS and cytokine production indicating that the *Ity3.1* locus when inherited from MOLF/Ei resulted in systemic innate immune deficiency leading to uncontrolled bacterial growth. The *Ity3.2* sublocus presented a hyper-responsive phenotype with respect to cytokine production during infection, a phenotype that can lead to septic shock and consequently increased susceptibility to infection. This hyperresponsive phenotype could not be detected when MOLF/Ei alleles are present at both *Ity3.1* and *Ity3.2* loci indicating the presence of interaction between these two loci.

We have previously reported no difference in bacterial burden between the mice carrying MOLF/Ei alleles at the *Ity3* locus and mice heterozygous (B6/MOLF) for the *Ity3* locus [9]. In this early study, the congenic mice were at generation N5 and we used heterozygous mice at the *Ity3* locus as controls because mice with the homozygous B6/B6 alleles at *Ity3* mice were not available. In addition, no difference in phenotypic expression was expected between *Ity3^B6/B6^* and *Ity3^MOLF/B6^* because of the recessive mode of inheritance of *Ity3* as established in the original F2 cross [8]. In the study by Sancho-Shimizu et al [9], [12] bacterial loads during infection were similar in *Ity3^MOLF/MOLF^* and *Ity3^MOLF/B6^* mice which contrast with the significant difference observed in the current study when *Ity3^MOLF/MOLF^* mice were compared to *Ity3^B6/B6^* suggesting that the transfer of *Ity3* onto a C57BL/6J background for more than 10 generations, results in a codominant mode of inheritance for the bacterial load phenotype. These observations highlight the complex nature of the expressivity of *Ity3* with respect to genetic background during infection with *Salmonella*.

Our study demonstrated that there was a sex-dependent effect in the outcome of infection with *Salmonella* in congenic mice, the male *Ity* strain being more resistant to infection than female *Ity* mice although males and females mice carrying *Ity3* presented the same degree of susceptibility. There is significant amount of research illustrating the fact that sex hormones exert potent effects on the immune system [18]–[20]. Previous studies in our laboratory have identified sex-specific QTLs in a chronic model of *Salmonella* persistence [21] and treatment of female mice with estradiol was shown to increase susceptibility to *Salmonella* infection, whereas treatment with progesterone increased resistance to infection [22].

The *Ity3* segment is a highly gene rich locus (272 protein coding genes and 107 non coding RNA) and this region of chromosome 1 harbours a large number of QTLs (56 QTLs are listed at MGI) influencing different aspects of metabolism and physiology, and immunological, cancer and behavioural traits. This QTL-rich region is also known to modulate the expression of many genes and phenotypes [11], [23]. The corresponding region in humans, Chr 1q21-q25, has been associated with neurobehavioural and metabolic traits (reviewed in [11]). Two additional QTLs directly involved in the host response to infection were mapped to the *Ity3* region, *Berr1* (berghei resistance locus 1) which confers resistance to cerebral malaria [24] and *Ssta2 (*susceptibility to *Salmonella* Typhimurium antigens 2) [25]. The *Ssta2* locus was mapped by intercrossing two mouse strains selected for high or low antibody response to flagellar antigens of *Salmonella*. The high producing mice were more susceptible to infection and showed reduced IFNγ levels in spleen homogenates, an observation that is consistent with the reduced IFNγ production in *Ity3* susceptible mice during infection.

In addition to highlighting the complex nature of the innate immune response driven by the *Ity3* locus, the current study further supports the hypothesis that reduced *Ncf2* activity leads to reduced activation of NF-κB signalling and bactericidal activity [26]. In addition to playing an active role in killing bacteria by producing toxic reactive oxygen intermediaries, ROS is a key signal modulator of TLR activated autophagy of phagosomes, and of caspase-1-induced pyropoptosis [27]–[29]. In fact, we did observe reduced IL-1β secretion and reduced cell death in BMDM of *Ity3*.*RecG* and *Ity3.RecN* strains suggesting that pyroptosis may be a bacterial killing mechanism affected by low ROS levels during *Salmonella* infection.

In summary, we have shown the existence of two distinct loci within *Ity3* that affect different aspects of the immune response and act together to confer susceptibility to *Salmonella* infection in MOLF/Ei mice. We provide additional evidence of the candidacy of *Ncf2* as the gene responsible for the *Ity3.1* locus. The validation of *Ncf2* as the gene underlying *Ity3.1* is awaiting the creation of a knock-in mouse model and the identification of the gene underlying *Ity3.2* will provide a better understanding of the genetic complexity of *Ity3*.
